# Down syndrome and parental depression: A double hit on early expressive language development

**DOI:** 10.1016/j.ridd.2020.103613

**Published:** 2020-05

**Authors:** Hana D’Souza, Amanda Lathan, Annette Karmiloff-Smith, Denis Mareschal

**Affiliations:** a Department of Psychology, University of Cambridge, United Kingdom; b Newnham College, University of Cambridge, United Kingdom; c Centre for Brain and Cognitive Development, Birkbeck, University of London, United Kingdom; d The LonDownS Consortium, United Kingdom

**Keywords:** Parental depression, Down syndrome, Cross-sectional developmental trajectories, Expressive language, Language development, MacArthur-Bates Communicative Development Inventory, Mullen Scales of Early Learning

## Abstract

**Background and aims:**

Down syndrome (DS) is often characterised by intellectual disability with particular difficulties in expressive language. However, large individual differences exist in expressive language across development in DS. In the general population, one of the factors associated with variability in this domain is parental depression. We investigated whether this is also the case in young children with DS.

**Methods:**

Thirty-eight children with DS between 8 and 48 months of age participated in this study. Their parents reported on the children’s receptive and expressive vocabularies (MacArthur-Bates Communicative Development Inventory) and on parental depression. Furthermore, an experimenter-led standardized developmental assessment (Mullen Scales of Early Learning) was administered to the children to test five domains: gross motor, fine motor, visual reception, receptive language, and expressive language.

**Results:**

A cross-sectional developmental trajectories analysis demonstrated that expressive language developed at a slower rate in children with DS whose parent reported depression than in those whose parent did not. No differences between groups were found in any other domain.

**Conclusion:**

Parental depression is associated with slower rate of expressive language development in young children with DS. These findings suggest that DS and parental depression may constitute a double hit leading to increased difficulties in the development of expressive language.

## Highlights

• Parental depression relates to slower rate of expressive language development in young children with Down syndrome.
• No association with parental depression was found for any other domain.
• Parental depression and Down syndrome may constitute a double hit on expressive language development.

## What this paper adds?

Little is known about the relationship between parental depression and the development of young children with Down syndrome (DS). We found that parental depression is associated with a slower rate of expressive language development in the first four years of life in DS. These findings suggest that DS and parental depression may constitute a double hit leading to increased difficulties in the development of expressive language. Thus, family systems with this double vulnerability may be a particularly important target for intervention.

## 1 Introduction

Down syndrome (DS) is caused by the presence of an additional chromosome 21. With an incidence of approximately 1 in 1000 live births, DS is the most common known genetic cause of intellectual disability (Wu & Morris, 2013). A characteristic profile of strengths and weaknesses has been described in DS, with particular difficulties in expressive language (Chapman, 1997; Daunhauer & Fidler, 2011). These difficulties are apparent early in development and become more pronounced over developmental time (Dykens, Hodapp, & Evans, 2006; Fidler, Hepburn, & Rogers, 2006; Miller, 1999). Infants with DS show a delay of around two months in the onset of canonical babbling compared to typically developing infants (Lynch et al., 1995). A delay of around 12 months has been reported for the ten word stage in children with DS (Oliver & Buckley, 1994). As language complexity increases, so does the delay, with two-word phrases showing a delay of around 18 months (Oliver & Buckley, 1994).

Although expressive language development is particularly poor in DS, large individual differences exist in this domain (as they do in other domains; Karmiloff-Smith et al., 2016). Zampini and D’Odorico (2009) carried out a longitudinal study of vocabulary development in 20 children with DS using the Italian version of the MacArthur Communicative Development Inventory. At 36 months of age, the lowest performing child with DS was non-verbal while the highest scoring child with DS was close to the typical range, producing 243 words. Six months later, the non-verbal child with DS remained non-verbal, while the expressive vocabulary of the highest scoring child with DS had doubled in size. Understanding the factors underlying large individual differences in expressive language in DS presents a crucial step towards future interventions (D’Souza, D’Souza, & Karmiloff-Smith, 2017; Karmiloff-Smith et al., 2016).

In the general population, one of the factors which has been systematically shown to negatively affect early cognitive, behavioural, and socio-emotional development is parental depression (see Ahun & Côté, 2019 for a review; Liu et al., 2017 for a meta-analysis). Moreover, depression is a significant problem for parents of young children, with 14% of mothers and 10% of fathers showing elevated depressive symptoms (Paulson, Keefe, & Leiferman, 2009). One of the first domains that is negatively affected by parental depressive symptoms is expressive language development. Maternal depression post-partum and at 12 months of age has been significantly associated with poor language outcomes at 12 months of age, measured using the language scale (receptive and expressive language combined) of the Bayley Scales of Infant Development III (Quevedo et al., 2012). When receptive and expressive language were assessed separately at 12 months of age, it was expressive language that was negatively associated with maternal depression (Kaplan et al., 2014). Stein et al. (1991) compared the 19-month-olds of mothers who had depressive disorder in the post-natal year with those without any psychiatric diagnosis since delivery. The children’s development was assessed across four domains: gross motor, language, fine motor-adaptive, and personal and social. The only difference between the groups was in the domain of expressive language. Specifically, fewer children could combine two words if their mother had depression.

Taken together, while parental depression has a negative impact on a range of domains in older children (Cicchetti, Rogosch, & Toth, 2000; Gueron-Sela et al., 2018; Kiernan & Huerta, 2008; NICHD Early Child Care Research Network, 1999; Stein, Malmberg, Sylva, Barnes, & Leach, 2008), expressive language appears to be one of the first domains negatively affected by parental depression in very young children. This domain also happens to be one with great vulnerability in children with DS. Thus, studying the effect of parental depression on expressive language in DS is of great importance, especially since mental health difficulties may be more frequent in parents of children with neurodevelopmental disorders, including DS (e.g., Hedov, Annerén, & Wikblad, 2000; Roach, Orsmond, & Barratt, 1999; Spangenberg & Theron, 2001). Yet, to our knowledge, no study has examined the link between parental depression and developmental outcomes in young children with DS. The current paper investigated the relationship between parental depression and the domain of greatest vulnerability in children with DS – expressive language. In order to examine whether this relationship is domain-specific, we also investigated associations between parental depression and other domains (receptive language, gross motor, fine motor, and visual reception).

## 2 Methods

### 2.1 Participants

Thirty-eight children with DS between 8 and 48 months, and their parents, participated in this study. The children with DS were selected from a large sample of children with DS (*N* = 115) within the London Down Syndrome (LonDownS) Consortium (for detailed demographics and health information, see Startin et al., 2020). Nineteen children from the LonDownS Consortium sample were included in the current study because their mother (*n* = 11), father (*n* = 5), or both parents (*n* = 3) reported a diagnosis of depression. These children (the 'Depression group') were closely matched to 19 children with DS whose parent(s) did not have depression (the 'No-depression group'; for participant characteristics, see Table 1). Matching factors included several parent-report factors: the child’s chronological age at time of questionnaire completion, the child’s gender, the child’s ethnicity, parental age, number of children and adults in the household, and socioeconomic status (parental education and occupation). Education levels were rated on a scale from secondary education to postgraduate degree, while occupations were classified using the UK Office of National Statistics standard occupational classification 2010. If education levels and/or occupation classifications differed between each child’s parents, then the highest level/classification was used.

**Table 1:** Participant characteristics.

		Group		Comparison
		Depression	No-depression	
**Number of children**		19	19	

**Child’s age (months)**		8-48 *M* = 31.2, *SD* = 12.5	8-43 *M* = 28.4, *SD* = 11.2	*t*(36) = 0.71, *p* = .482

**Child’s gender**	Female	9	7	*χ^2^*(1) = 0.43, *p* = .743
	Male	10	12	

**Child’s ethnicity**	White	17	15	Fisher’s exact test = 4.29 *p* = .169
	Black	0	0	
	Asian	0	2	
	Mixed	2	0	
	Other	0	1	
	Missing	0	1	

**Mother’s age (years)**		31-48 (1 missing) *M* = 41.3, *SD* = 4.9	28-48 (3 missing) *M* = 38.6, *SD* = 6.0	*t*(32) = 1.49, *p* = .147

**Father’s age (years)**		36-53 (0 missing) *M* = 42.6, *SD* = 5.2	34-59 (4 missing) *M* = 43.3, *SD* = 5.9	*t*(32) = 0.36, *p* = .720

**Number of children in household**		1-4 *Mdn* = 2.0, *IQR* = 1.0-2.0	1-3 *Mdn* = 2.0, *IQR* = 2.0-2.0	*U* = 137.50, *p* = .212

**Number of adults in household**		2 *Mdn* = 2.0, *IQR* = 2.0-2.0	1-2 *Mdn* = 2.0, *IQR* = 2.0-2.0	*U* = 199.50, *p* = .583

**Parental education**	Postgraduate degree	5	6	Fisher’s exact test = 1.85 *p* = .860
	Undergraduate degree	7	9	
	Vocational/College	3	1	
	A-levels	2	1	
	Secondary education	2	2	

**Parental occupation**	Managers, directors and senior officials	5	6	Fisher’s exact test = 3.64 *p* = .769
	Professional occupations	8	6	
	Associate professional and technical occupations	3	5	
	Administrative and secretarial occupations	0	1	
	Skilled trades occupations	1	0	
	Caring, leisure and other service occupations	0	0	
	Sales and customer service occupations	0	1	
	Process, plant and machine operatives	0	0	
	Elementary occupations	0	0	
	Missing	2	0	

The participants were recruited via existing participant databases and support groups. Ethical approval was obtained from the North West Wales National Health Service (NHS) Research Ethics Committee (13/WA/0194) and from the Birkbeck Psychological Sciences Ethics Committee (121373). Prior to testing, informed consent was obtained from parents. Participants were given a small gift (e.g., a T-shirt) in return for their participation.

### 2.2 Procedure and materials

#### 2.2.1 Parental report of depression

Parents were administered a medical history questionnaire over the phone before the lab visit (Startin et al., 2020). They were asked whether either parent had ever been diagnosed with depression. A dichotomous variable yes/no was generated based on their responses which differentiated the children with DS into a Depression group and a No-depression group.

#### 2.2.2 MacArthur-Bates Communicative Development Inventory

Parents filled in the MacArthur-Bates Communicative Development Inventory (CDI) for Words and Gestures (for ages 8-18 months) (Fenson, Marchman, Thal, Dale, & Reznick, 2007). Although the age range of our participants with DS was 8-48 months, the CDI Words and Gestures was an appropriate measure considering the language delay in DS (Zampini & D’Odorico, 2009). The parent reported on their child’s ability to understand and/or say words from the checklist. The present study used items from the Vocabulary section of the CDI to measure receptive and expressive vocabulary size. Forms of sign language such as Makaton were included in parental reports and scoring. In the Depression group, all questionnaires were completed by the child’s mother (*n* = 19*)*, of whom 14 had a diagnosis of depression at some point in their life. In the No-depression group (*n* = 19*)*, 16 mothers, 2 fathers, and in one case both parents together completed the questionnaire.

#### 2.2.3 Mullen Scales of Early Learning

The Mullen Scales of Early Learning (MSEL; Mullen, 1995) was administered by an experimenter at the lab visit. MSEL is a standardized assessment which comprises five domains: (1) *gross motor* (central motor control and mobility in supine, prone, sitting, and fully upright positions); (2) *fine motor* (visually-directed motor planning, object manipulation, visual discrimination (motor planning), and motor control); (3) *visual reception* (visual perceptual ability, spatial awareness, and visual memory); (4) *receptive language* (auditory comprehension, auditory memory, and the ability to process linguistic input); and (5) *expressive language* (the ability to use sounds and language productively). These yield scores for each domain. MSEL is standardized for TD children between 0 and 68 months (from 0 to 33 months for the gross motor domain). This test has high internal consistency and test-retest reliability (Mullen, 1995; see also Bishop, Guthrie, Coffing, & Lord, 2011, for estimates of convergent validity). Following the previous literature (Dykens et al., 2006; Marchal et al., 2016), we used age equivalents rather than standard scores for data analysis, because standard scores are often at floor. The MSEL was completed within one month of completion of the CDI – with the exception of five participants (Depression group: within two months (*n* = 2), within five months (*n* = 1); No-depression group: within two months (*n* = 1), within three months (*n* = 1)). Analyses of the relationship between MSEL and CDI were run both with and without these five participants, to ascertain whether this affected the results.

### 2.3 Cross-sectional developmental trajectory analysis

In order to investigate whether changes in scores differ across groups (Depression, No-depression) and chronological age, we employed a cross-sectional developmental trajectory analysis (Thomas et al., 2009; see also Annaz, Karmiloff-Smith, Johnson, & Thomas, 2009; Jarrold, Baddeley, & Phillips, 2007; Purser et al., 2015; Thomas, Purser, & Van Herwegen, 2011). The method uses Analysis of Covariance (ANCOVA). However, whereas a typical ANCOVA would be employed to test for differences between groups while “accounting for” chronological age, the cross-sectional developmental trajectories analysis utilises the covariate (chronological age) itself. It evaluates differences between regression lines which depict the developmental trajectories of outcome variables in the two groups across chronological age. While for a typical ANCOVA analysis, the average performance of each group is represented by a single number (the mean), two numbers represent cross-sectional developmental trajectories: the *intercept* and *gradient*. The intercept describes the initial level of performance. The gradient represents the rate at which performance changes (increases or decreases) with age. The use of intercepts and gradients allows us to look beyond differences between means (which can only inform us of *whether* performance differs across groups or not) and provides insight into *how* performance differs in the two groups across developmental time.

In this paper, cross-sectional developmental trajectories were initially constructed separately for each group by conducting a series of linear regression analyses to examine whether chronological age relates to performance on an outcome variable. Subsequently, the cross-sectional developmental trajectories were compared between the Depression and No-depression groups. This made it possible to evaluate whether the developmental trajectories differed between the groups in terms of their gradients and/or intercepts. To compare pairs of trajectories, a modified version of ANCOVA was employed to test for a main effect of Chronological age, Group, and the interaction between Chronological age and Group. If the main effect of Group is significant, then the intercepts of the two groups are significantly different. We could then conclude that the performance of one group exhibits *delayed onset* in development compared to the other group. If the interaction between Chronological age and Group is significant, then we can conclude that one group was developing more slowly than the other, exhibiting a slower *rate* of development.

## 3 Results

**Fig. 1:**
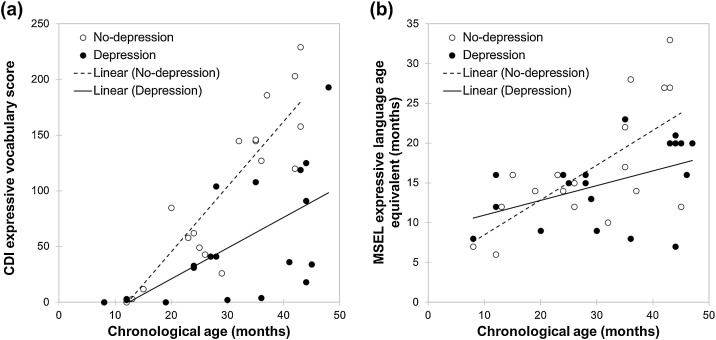
Cross-sectional developmental trajectories comparing children with DS with and without parents who reported having had a diagnosis of depression (the Depression and No-depression groups, respectively) on (a) CDI expressive vocabulary score plotted against chronological age (months); and (b) MSEL expressive language age equivalent (months) plotted against chronological age (months).

Fig. 1a shows developmental trajectories relating Chronological age and CDI expressive language in the Depression and No-depression groups. A significant relationship between Chronological age and CDI expressive language was present in both the Depression group (*R^2^* = .39, *F*(1,17) = 10.81, *p* = .004) and No-depression group (*R^2^* = .80, *F*(1,17) = 68.64, *p* < .001). The Depression group trajectory plotted against Chronological age was compared with the No-depression group trajectory using a general linear model predicting overall CDI expressive language from Chronological age with Group as a between-subject factor. As expected, there was a significant main effect of Chronological age, *F*(1,34) = 59.80, *p* < .001, η_p_^2^ = .64, which indicates that across both groups, Chronological age significantly predicted CDI expressive language. The main effect of Group was not significant, *F*(1,34) = 1.12, *p* = .298, η_p_^2^ = .03. This indicates that the intercepts of the Depression and No-depression groups were not significantly different. However, the gradient was different between the two groups as indicated by a significant interaction between Group and Chronological age, *F*(1,34) = 7.69, *p* = .009, η_p_^2^ = .19. Thus, the Depression group showed a slower rate of development than the No-depression group.

To check that the finding for our parent report measure of expressive language also exists for our *experimenter-led* measure, we conducted the same developmental trajectory analysis as before – but with MSEL expressive language. First, we established the relationship between parent-reported CDI expressive language and experimenter-led MSEL expressive language (*r*(35) = .57, *p* < .001, chronological age at CDI partialled out). A near-identical result was obtained when chronological age at MSEL was partialled out (*r*(35) = .58, *p* < .001) or when the correlation was run only with participants for whom CDI and MSEL were administered within one month of each other (*r*(30) = .60, *p* < .001, chronological age at CDI/MSEL partialled out).

We then conducted the cross-sectional developmental trajectory analysis with MSEL expressive language (Fig. 1b). A significant relationship between Chronological age and MSEL expressive language was present in both the Depression group (*R^2^* = .22, *F*(1,17) = 4.69, *p* = .045) and No-depression group (*R^2^* = .46, *F*(1,17) = 14.26, *p* = .002). The Depression group trajectory plotted against Chronological age was compared with the No-depression group trajectory using a general linear model predicting overall MSEL expressive language from Chronological age with Group as a between-subject factor (Fig. 1b). As expected, there was a significant main effect of Chronological age, *F*(1,34) = 19.06, *p* < .001, η_p_^2^ = .36, which indicates that across both groups, Chronological age significantly predicted MSEL expressive language. The main effect of Group was not significant, *F*(1,34) = 1.20, *p* = .282, η_p_^2^ = .03. This indicates that the intercepts of the Depression and No-depression groups were not significantly different. The interaction between Group and Chronological age approached significance, *F*(1,34) = 3.12, *p* = .086, η_p_^2^ = .08. This is broadly consistent with the above finding that expressive language (CDI) was developing at a slower rate in the Depression group than the No-depression group.

The relationship between parental depression and expressive language in DS may be domain-specific because parental depression was not linked to any other outcome measure (CDI receptive language, MSEL gross motor, MSEL fine motor, MSEL visual reception, MSEL receptive language). While comparisons of group trajectories found significant main effects of Chronological age on the outcomes (all *F*s(1,33/34) > 47.38, *p*s < .001, η_p_^2^s > .58), there were neither main effects of Group (all *F*s(1,33/34) < 0.78, *p*s > .384, η_p_^2^s < .03) nor Group x Chronological age interactions (all *F*s(1,33/34) < 2.68, *p*s > .111, η_p_^2^s < .08). Therefore, the Depression and No-depression groups did not significantly differ from each other on CDI receptive language, MSEL gross motor, MSEL fine motor, MSEL visual reception, or MSEL receptive language.

## 4 Discussion

To our knowledge, we have demonstrated for the first time an association between parental depression and a slower rate of expressive language development in the first four years of life in DS. Importantly, the relationship seems to be specific for expressive language – as no other measure showed an association with parental depression. Although the current paper cannot provide us with a mechanistic account of this relationship, it highlights the importance of understanding development in groups with multiple vulnerabilities (parental depression and DS) as these groups may have a greater risk of developing poor outcomes.

One of the pathways which could explain the relationship between parental depression and expressive language is through the context of parent-child interaction (for a review, see Ahun & Côté, 2019). Parents’ depressive symptoms have been associated with difficulties in responding in a sensitive and consistent manner to their child due to preoccupation with symptoms, fatigue, poor concentration, and/or agitation (for a meta-analytic review, see Lovejoy, Graczyk, O’Hare, & Neuman, 2000; for a review, see Murray, Cooper, & Hipwell, 2003). Characteristics of parent-child interaction present in parents with depression have been linked to poorer developmental outcomes in children across a range of domains, including language (for reviews, see Ahun & Côté, 2019; Sohr-Preston & Scaramella, 2006).

The interaction between a parent and child provides an important context for the child’s expressive language development – a domain in which children with DS particularly struggle (Chapman, 1997; Daunhauer & Fidler, 2011). Parental stimulation may be especially important for children with DS in this domain as these children seem to vocalize less than typically developing children of the same chronological age (Thiemann-Bourque, Warren, Brady, Gilkerson, & Richards, 2014). Yet, it may be difficult for parents to stimulate their child with DS if they have depression. In typically developing children, mothers with depression were found to engage less in activities which promote language development: they use less infant-directed speech, participate less in facial, vocal, and play interaction, are less focused on infant experience, and provide fewer stimulating activities than mothers without depression (Kaplan, Burgess, Sliter, & Moreno, 2009; Righetti-Veltema, Bousquet, & Manzano, 2003; Stein et al., 1991; Zajicek-Farber, 2010; for reviews, see Field, 2010; Sohr-Preston & Scaramella, 2006). Additionally, parents with depression are more likely to express hostility and negative affect (for reviews, see Downey & Coyne, 1990; Field, 2010). Children’s exposure to stimulating activities mediated the effect of parenting, and maternal mental health predicted expressive and receptive vocabulary in the second year of life (Zajicek-Farber, 2010). Similarly, Stein et al. (2008) reported that post-natal depression negatively affected caregiving, which in turn negatively affected language development (expressive and receptive language combined) measured at 36 months of age. It is possible that parent-child interaction also mediates the relationship between parental depression and expressive language in children with DS we observed in the current study.

Considering that parent-child interaction is an important context for learning more broadly, it is unlikely that parental depression solely impacts expressive language. Indeed, findings from studies of typically developing children show that even though the expressive language domain is often the first domain to be negatively impacted by parental depression (Kaplan et al., 2014), perhaps because parent-child interaction is particularly important for expressive language development, over developmental time parental depression affects multiple domains (for a review, see Ahun & Côté, 2019). It is possible that our sample of children with DS was either too young in age or too small in number to detect the effects of parental depression on other domains. Perhaps in the case of neurodevelopmental disorders, parental depression increases vulnerability to poor outcomes across a range of domains, but the effect of depression may be especially pronounced (and thus detectable with a small sample size) in the domain that the particular population struggles with the most. In the case of DS, that domain is expressive language (Chapman, 1997; Daunhauer & Fidler, 2011). In other neurodevelopmental disorders, however, the most affected domain may differ. Future cross-syndrome comparisons could provide us with important insights into these relationships.

It is important to emphasize that the effect of parental depression may not necessarily be an environmental effect. Difficulties in expressive language in children with DS may be due to an inherited genetic vulnerability – which in parents would be associated with depression and in infants/toddlers with DS exacerbate the difficulties in the domain with the highest vulnerability – expressive language. Future studies could investigate these questions as well as possible gene-environment correlations and gene-environment interactions (Natsuaki et al., 2010).

There are some limitations of the current study which are important to consider. The main result of the effect of depression on CDI expressive language was based on a parental report of depression and expressive language. It is possible that parents who report depression also report more negatively on their child’s performance (Ordway, 2011). However, several results in the current paper suggest otherwise. Firstly, if the parents who had been diagnosed with depression reported more negatively on their children, we would expect to find this bias across different measures, and perhaps especially in measures that may allow for the most subjective interpretation – such as receptive language. However, this did not seem to be the case as the observed effect was specific to expressive language. Secondly, the result of expressive language from the parental report was in line with results showing a trend in the expressive language scale of the experimenter-led standardized test (MSEL). The reason MSEL expressive language only approached significance may be due to differences between the CDI and MSEL expressive language measures. The CDI vocabulary section assesses the vocabulary of the child through a checklist and included any sign language. In contrast, the expressive scale of the MSEL assesses spoken expressive language more broadly – including vocalizations, babbling, gestures, and word production. The contrast between these two measures in the current study seems to suggest that parental depression mostly affects the ability to produce words in this particular age group. Furthermore, considering all MSEL measures, the trend was only observed for expressive language, with no differences reported for any other MSEL scale. This is in line with results from the parental report.

Another limitation of this study is that it is based on a convenience sample. It is possible that our sample does not represent those parents with depression who find it particularly challenging to deal with their child with DS, as these parents may find it difficult to allocate time and other resources to take part in research studies. Thus, it is possible that the current study actually underestimates the impact of parental depression on development in children with DS, since in studies of TD children, severe parental depression has been found to have greater negative effects on development (Brennan et al., 2000). Furthermore, our participants were mostly from relatively high socioeconomic backgrounds. A more representative sample may shed light on the additional challenges some families of children with DS face and how it affects the children’s development.

In order to understand the link between parental depression and language outcomes in children with DS, future studies should also obtain more detailed information on parental depression, such as onset, duration, and severity, as these have been related to developmental outcomes in typically developing children (Brennan et al., 2000; Quevedo et al., 2012; Sohr-Preston & Scaramella, 2006). Obtaining more detailed information on the timing of depression would allow us to unpack potential differential effects of cases in which a diagnosis of depression precedes (versus follows) the birth of the child with DS. It would also enable us to understand the extent to which the DS diagnosis, level of difficulties a particular child with DS faces, and potential comorbidities (Visootsak et al., 2016) may contribute to the onset and/or severity of depression in the parent. Future research in DS also needs to differentiate the effects of post-partum depression from other types of depression (Korhonen, Luoma, Salmelin, & Tamminen, 2012), and the effects of paternal versus maternal depression (Paulson et al., 2009; Ramchandani, Stein, Evans, & O’Connor, 2005). Furthermore, depression medication during pregnancy may affect language development (Weikum, Oberlander, Hensch, & Werker, 2012). Many other factors are likely to affect the relationship between parental depression and language development. For example, language development has been linked to both siblings (e.g., Bridges & Hoff, 2014) and sleep (e.g., D’Souza, D’Souza, Horváth, Plunkett, & Karmiloff-Smith, 2020). All these effects remain to be studied in children with DS using bigger samples and longitudinal designs.

This study represents an important step towards understanding how parental depression relates to developmental outcomes in children with DS. Although a number of studies have investigated the impact of parenting on development in DS (for reviews, see Daunhauer, Schworer, & Howshar, 2017; Te Kaat-van den Os, Jongmans, Volman, & Lauteslager, 2017) as well as the psychological health of parents of children with DS and other neurodevelopmental disorders (Hedov et al., 2000; Roach et al., 1999; Spangenberg & Theron, 2001), we need studies that bridge between the two topics; i.e., understanding how the psychological health of parents of children with DS affects their child’s developmental outcomes. This clearly seems to be an important subject to address as the current study shows that children with DS who also have a parent who has experienced depression particularly fall behind in their expressive language. Thus, family systems with a double vulnerability (DS and parental depression) may be an especially important target for intervention.

## CRediT authorship contribution statement

**Hana D’Souza:** Conceptualization, Data curation, Formal analysis, Funding acquisition, Investigation, Methodology, Project administration, Supervision, Visualization, Writing - original draft, Writing - review & editing. **Amanda Lathan:** Conceptualization, Data curation, Formal analysis, Methodology, Visualization, Writing - original draft, Writing - review & editing. **Annette Karmiloff-Smith:** Conceptualization, Funding acquisition, Methodology, Project administration, Resources, Supervision. **Denis Mareschal:** Project administration, Resources, Supervision, Writing - review & editing.

## Declaration of Competing Interest

None.
